# Parental Firearm Storage and Their Teens’ Perceived Firearm Access in US Households

**DOI:** 10.1001/jamanetworkopen.2025.14443

**Published:** 2025-06-10

**Authors:** Katherine G. Hastings, Patrick M. Carter, Marc Zimmerman, Rebeccah Sokol

**Affiliations:** 1 Institute for Firearm Injury Prevention, University of Michigan, Ann Arbor; 2 School of Population and Public Health, University of British Columbia, Vancouver, Canada; 3 Department of Emergency Medicine, School of Medicine, University of Michigan, Ann Arbor; 4 Health Behavior and Health Education, School of Public Health, University of Michigan, Ann Arbor; 5 School of Social Work, University of Michigan, Ann Arbor

## Abstract

**Importance:**

Firearm access increases fatal and nonfatal firearm injury risk among teens. Identifying parental firearm storage behaviors associated with teen access may inform efforts to prevent teen firearm injuries.

**Objective:**

To examine the associations between parent-reported household firearm storage behaviors and teen perceived access to firearms.

**Design, Setting, and Participants:**

This cross-sectional study used national survey data of US firearm-owning parents and their teens (aged 14 to 18 years) surveyed between June and July 2020, with a response rate of 31% for parents and 21% for teens. Analyses were conducted January to May 2024.

**Exposures:**

The number of firearms stored in the household as (1) unlocked, (2) loaded, (3) unlocked and loaded, and (4) unlocked or loaded.

**Main Outcomes and Measures:**

Teen perceived firearm access, overall and stratified by teen gender, parental education, and urbanicity. Survey-weighted logistic regression analyses assessed the associations between the number of firearms parents reported storing in each unsecured state (unlocked and loaded, unlocked, loaded, and unlocked or loaded) and teen perceived firearm access, overall and stratified by teen gender, parental education, and urbanicity (metropolitan vs nonmetropolitan). The areas under the receiver operating characteristic curves (AUROC) were used to identify the firearm storage behaviors with the best ability to estimate teen perceived firearm access.

**Results:**

Analyses included 487 parent-teen dyad respondents. The mean (SE) ages of parents and teens were 46.6 (0.80) and 16.0 (0.12) years, respectively. Most parent respondents were male (58.1%; 95% CI, 50.3%-65.8%) and White (73.5%; 95% CI, 66.1%-80.9%), and most teen respondents were female (55.7%; 95% CI, 47.8%-63.6%) and White (69.5%; 95% CI, 61.8%-77.2%). All 4 unsecured firearm storage behaviors were associated with greater teen perceived firearm access (odds ratio [OR], 1.27-1.44; 95% CI, 0.99-2.10), but associations disappeared after restricting to those who stored at least 1 firearm unsecured (OR, 0.99-1.18; 95% CI, 0.67-1.89). The number of firearms stored unlocked performed the best in estimating teen perceived access to firearms in US households (AUROC, 65.7; 95% CI, 61.4-70.1), regardless of teen gender, parental education, and urbanicity. However, sensitivity of this measure was universally low (range, 42%-64%). Additionally, 36.3% (95% CI, 23.6%-49.0%) of teens reported access to a firearm in households where all firearms were stored locked and unloaded.

**Conclusions and Relevance:**

This study found that parent-reported firearm storage may be a poor estimator of teen perceived firearm access, regardless of teen gender, parental education, and urbanicity. Strictly focusing safety efforts on locked and unloaded firearm storage may not fully negate teen’s perceptions that they can access and load household firearms. Storing additional firearms securely may not prevent teen access if at least 1 household firearm remains unlocked.

## Key Points

**Question:**

What are the associations between the number of household firearms stored in different behaviors (ie, unlocked or locked, loaded or unloaded) and teens’ perceived access to firearms?

**Findings:**

In this cross-sectional study of 487 parent-teen dyads, parent-reported firearm storage poorly estimated teens’ perceived firearm access, regardless of teen gender, urbanicity, and parental education. Any additional securely stored firearms were not associated with teen perceived access if at least 1 firearm remained unlocked in the household.

**Meaning:**

These findings suggest that efforts to prevent teen firearm injuries should consider more comprehensive approaches to firearm access prevention in addition to securely storing firearms.

## Teaser

This cross-sectional study examines the associations between parent-reported household firearm storage behaviors and teen perceived access to firearms using national survey data.

## Introduction

Firearms are the leading cause of death among children and teens (ages 1 to 19 years) in the US.^1,2^ Most firearm-involved deaths among youth happen with a firearm from their home or from that of a friend or family member.^3,4,5^ Preventing these deaths requires reducing unsupervised teen access to firearms in their own homes and the homes where they spend time.

Many recommendations, interventions, and policies to prevent teen access to firearms focus on household firearm storage, encouraging adult firearm owners to securely store firearms locked and unloaded.^6,7^ Prior research supports these efforts, demonstrating that storing household firearms securely (eg, locked and unloaded) is associated with fewer firearm injuries and deaths for youth, as compared with storing firearms less securely.^8,9^ Studies have also shown that practicing triple secure storage—keeping firearms locked, unloaded, and storing ammunition separately—significantly reduces fatality risks across all age groups, firearm types, and injury causes.^9,10^ To date, few studies have examined associations between these specific household firearm storage behaviors and the likelihood of youth access.^11^ Given the salience of firearm storage behaviors as a primary prevention mechanism, it is critical to understand whether and which of these storage behaviors are associated with perceived youth firearm access.

Most firearm owners in the US own multiple firearms per household.^12^ Here, we test 2 competing hypotheses regarding the association between storage behaviors of these multiple firearms and youth firearm access. The first hypothesis, the harm reduction hypothesis, is that storing a greater proportion of household firearms securely (eg, locked and unloaded) reduces the likelihood of perceived youth firearm access, even if 1 firearm remains unsecured (eg, unlocked or loaded). The second hypothesis, the harm elimination hypothesis, is that storing a greater proportion of household firearms securely will not reduce the likelihood of perceived youth firearm access if at least 1 firearm remains unsecured; only 1 unsecured firearm is sufficient to increase injury risk among youth. Identifying which of these mechanisms govern youth firearm access will help advance policy and practices to improve firearm safety and reduce youth injury and death.

To test the harm reduction and harm elimination hypotheses, we examined associations between parent-reported firearm storage behaviors and teen perceived access to a firearm and an ability to load it (herein, teen perceived firearm access) among a nationally representative sample of US parents who own firearms and their teenaged children. Additionally, we investigated if associations between firearm storage behaviors and teen perceived firearm access varied by teen gender, parental education, and urbanicity. Findings from this study will help inform evidence-based interventions to mitigate the risks associated with firearm access among teens.

## Methods

### Data Source

Data obtained for this study came from the Firearm Safety Among Children and Teens National Parent-Teen Survey,^13^ a cross-sectional, web-based, and nationally representative survey of US parents and their teens aged 14 to 18 years regarding firearm-related practices. Data collection occurred from June 24 to July 22, 2020, using a Gallup panel, a probability-based panel recruited using random digit-dial phone interviews and address-based sampling methods constructed to be broadly representative of the US adult population. To minimize variance due to weighting and to account for anticipated nonresponse by demographic group, the survey statisticians drew a stratified sample. Demographic distribution of the stratified sample matched the US population targets for US adults obtained from the 2017 Current Population Survey.

The survey drew an original sample of 11 179 parents aged 18 years and older; however, the exact age of the child was unknown at the time. Parents included primary caregivers of teens (eg, parent, stepparent, grandparent), which yielded a total of 2942 parent respondents of the original sample (31% response rate). Respondents’ teenaged children living in the household were also invited to complete a separate survey. Of this parent-teen dyad cohort, 2000 teen respondents completed the survey (21% response rate). Response rates include a denominator of many parents who may not have qualified based on child age, so response rates are conservative estimates.

All respondents provided informed consent and received a $5 incentive upon survey completion. The University of Michigan institutional review board reviewed and approved the use of these data for this study. The study followed the Strengthening the Reporting of Observational Studies in Epidemiology (STROBE) reporting guideline.^14^

### Study Population

The study population included parent-teen dyads, which included any parent respondents who also had a teen respondent aged 14 to 18 years living in the household (at least part-time) at the time of survey completion. Of the 2942 parents respondents and 2000 teen respondents who completed survey responses, we limited our study population to include only parents who owned and stored firearms on their property (n = 788). We further excluded any teen respondents who reported having a firearm that personally belonged to them (n = 168), parent-teen dyads with incomplete or missing firearm storage data (n = 42), and parent-teen dyads with incomplete or missing information on teen firearm access (n = 91). The final analytical sample included 487 parent-teen dyads. eFigure in Supplement 1 illustrates the study flowchart.

### Measures

#### Exposures

The primary exposure variables included 4 parent-reported unsecured firearm storage behaviors, including the number of firearms stored: (1) unlocked and loaded, (2) unlocked (regardless of loaded status), (3) loaded (regardless of locked status); and, (4) unlocked or loaded. Number of firearms in respective storage behaviors included both any handguns or long guns as reported by parents. eTable 1 in Supplement 1 contains full question and response wording for parent-reported analytic variables.

#### Outcome

The primary outcome was teen perceived firearm access. Teens responded to the survey item: “How long would it take you to get 1 of the guns on your property and load it if it wasn’t already loaded?”, with response options including less than 5 minutes, less than an hour, less than 2 hours, more than 2 hours, and could not get access. In this case, teen perceived access refers to both access and an ability to load the firearm. Firearm access was dichotomized for analysis into firearm access (response options: less than 5 minutes, less than an hour, less than 2 hours, more than 2 hours) vs no firearm access (response options: could not get access). eTable 2 in Supplement 1 contains full question and response wording for teen-reported analytic variables. Sensitivity analyses operationalized teen perceived firearm access as access in less than 2 hours and access in less than an hour.

#### Demographic Variables

Parental and teen demographic characteristics included age, gender, and race and ethnicity. All of these variables are self-reported by both parents and teen respondents in the respective surveys. Additionally, parents reported their highest level of education and zip code. Zip code data were used to categorize the primary home location as metropolitan or nonmetropolitan according to the Rural-Urban Commuting Areas (RUCA); as with prior research, the team categorized RUCA value of 1 as metropolitan and RUCA values of 2 to 10 as nonmetropolitan areas.^15^

### Statistical Analysis

Survey-weighted logistic regression analyses assessed the associations between the number of firearms parents reported storing in each unsecured state (unlocked and loaded, unlocked, loaded, and unlocked or loaded) and teen perceived firearm access, overall and stratified by teen gender, parental education, and urbanicity (metropolitan vs nonmetropolitan). We compared areas under the receiver operating characteristic curves (AUROC) to identify the firearm storage behaviors with the best ability to estimate teen perceived firearm access. We also used the AUROC curves to select the optimal threshold for categorization: the number of firearms stored in a specific manner that classified a teen as having perceived access vs no perceived access. We selected this optimum threshold based on the number of firearms in a storage behavior that maximized the distance of the curve to the identity. To investigate if risk for teen perceived firearm access increased with storing more firearms unsecured, additional analyses estimated the associations between the number of firearms parents reported storing in each unsecured manner and teen-reported firearm access among subsamples that reported storing 1 or more firearms in each respective manner.

Sensitivity analyses assessed the associations between the number of firearms parents reported storing in each unsecured storage behavior and perceived teen firearm access in less than 2 hours and less than 1 hour. In accordance with the American Statistical Association, inferences rely on the size on point estimates and width of 95% CIs rather than strictly *P* values.^16^ The team conducted all analyses in R version 4.3.1 (R Project for Statistical Computing). Data were analyzed from January to May 2024.

## Results

Among the 487 parent-teen dyads, the mean (SE) parental age was 46.6 (0.80) years, and most parent respondents were male (58.1%; 95% CI, 50.3%-65.8%) and attained an associate degree or less (69.1%; 95% CI, 63.1%-75.2%). Of parents who owned firearms, 70.0% (95% CI, 63.0%-77.1%) stored 1 or more firearms unlocked or loaded, 51.8% (95% CI, 44.0%-59.6%) stored 1 or more firearms loaded, 47.8% (95% CI, 40.0%-55.7%) store 1 or more firearms unlocked, and 24.8% (95% CI, 17.8%-31.7%) stored 1 or more firearms unlocked and loaded. The mean (SE) age of teen respondents was aged 16.0 (0.12) years, and most teen respondents were female (55.7%; 95% CI, 47.8%-63.6%), and lived in a metropolitan area (65.4%; 95% CI, 57.7%-73.0%). Of teen respondents, 52.1% (95% CI, 44.3%-60.0%) reported being able to access and load a household firearm. Table 1 and Table 2 provides detailed descriptive statistics for parent and teen respondents, respectively.

**Table 1.** Demographic Characteristics and Firearm Storage Behaviors Among Adult Firearm Owners of Teen Children in the US (n = 487)^a^

Demographic characteristics	Participants, No. (%)	(95% CI), %
Age, y	46.6 (0.80)	NA
Gender		
Male	336 (58.1)	(50.3-65.8)
Female	243 (41.9)	(34.1-49.7)
Race or ethnicity		
Asian	Suppressed	Suppressed
Black	71 (12.3)	(6.50-18.2)
Hispanic	80 (13.8)	(8.04-19.6)
White	426 (73.5)	(66.1-80.9)
Another race not listed	Suppressed	Suppressed
Education level		
Associate degree or less	400 (69.1)	(63.1-75.2)
4-y college degree or higher	179 (30.9)	(24.9-36.9)
Household firearm storage behaviors^b^		
Stores 1 or more firearms unlocked	277 (47.8)	(40.0-55.7)
Stores 1 or more firearms loaded	300 (51.8)	(44.0-59.6)
Stores 1 or more unlocked and loaded	144 (24.8)	(17.8-31.7)
Stores 1 or more unlocked or loaded	406 (70.0)	(63.0-77.1)
No. of firearms unlocked, mean (SD)	1.28 (0.15)	NA
No. of firearms loaded, mean (SD)	1.22 (0.14)	NA
No. of firearms unlocked and loaded, mean (SD)	0.62 (0.12)	NA
No. of firearms unlocked or loaded, mean (SD)	1.83 (0.15)	NA

Abbreviation: NA, not applicable.

^a^ Estimates weighted to be nationally representative. Counts derived from sample, and percentages derived from population estimates. The table suppresses data where cell counts are nonzero and less than 10 and data that enable inferring exact nonzero cell counts <10. We present race and ethnicity categories according to survey format.

^b^ Firearm storage behavior is not mutually exclusive across categories for each parent-respondent household.

**Table 2.** Demographic Characteristics and Perceived Firearm Access Among Teens (Age 14 to 18 years) of Adult Firearm Owners in the US (n = 487)^a^

Demographic characteristics	Counts, No. (%)	(95% CI)
Age, y	16.0 (0.12)	NA
Gender		
Male	243 (43.3)	(35.4-51.2)
Female	313 (55.7)	(47.8-63.6)
Gender expansive	Suppressed	Suppressed
Race		
White	390 (69.5)	(61.8-77.2)
Black	60 (10.7)	(4.9-16.6)
Asian	Suppressed	Suppressed
Multiracial	73 (13.0)	(7.6-18.3)
American Indian or Alaska Native	Suppressed	Suppressed
Native Hawaiian or Pacific Islander	Suppressed	Suppressed
Unknown	Suppressed	Suppressed
Hispanic ethnicity		
Hispanic, Latino, or Spanish origin	108 (19.3)	(12.6-26.0)
Not Hispanic, Latino, nor Spanish origin	452 (80.7)	(74.0-87.4)
Locale		
Metropolitan area	379 (65.4)	(57.7-73.0)
Nonmetropolitan area	200 (34.6)	(27.0-42.2)
Perceived firearm access		
Teen perceived firearm access		
Firearm access	302 (52.1)	(44.3-60.0)
No firearm access	277 (47.9)	(40.0-55.7)

Abbreviation: NA, not available.

^a^ Estimates weighted to be nationally representative. Counts derived from sample, and percentages derived from population estimates. The table suppresses data where cell counts are nonzero and <10 and data that enable inferring exact nonzero cell counts <10. We present race and ethnicity categories according to survey format.

In the unstratified sample, all 4 unsecured storage behaviors were positively associated with teen perceived firearm access in the full sample. Specifically, analyses show that the number of firearms stored unlocked (odds ratio [OR], 1.27; 95% CI, 1.02-1.58), loaded (OR, 1.28; 95% CI, 1.03-1.58), unlocked and loaded (OR, 1.44; 95% CI, 0.99-2.10), or unlocked or loaded (OR, 1.29; 95% CI, 1.06-1.56) were associated with an increased teen perception that they could access firearm(s) (Table 3).

**Table 3.** Comparison of the Accuracy of Parent-Reported Firearm Storage for Estimating Teen Perceived Firearm Access Among a Nationally Representative Sample of Adult Firearm Owners and Their Teen Children (n = 487)^a^

Firearm storage	OR (95% CI)	AUROC (95% CI)	*P* value	Optimum threshold
Method A, No. of firearms unlocked	1.27 (1.02-1.58)	65.7 (61.4-70.1)	<.01 vs B^b^	1 Firearm unlocked: sensitivity = 58.0%; specificity = 72.0%
			<.01 vs C^b^	
			.03 vs D^b^	
Method B, No. of firearms loaded	1.28 (1.03-1.58)	54.4 (49.7-59.1)	<.01 vs A^b^	2 Firearms loaded: sensitivity = 32.4%; specificity = 76.9%
			.03 vs C^b^	
			<.01 vs D^b^	
Method C, No. of firearms unlocked and loaded	1.44 (0.99-2.10)	58.5 (55.1-62.0)	<.01 vs A^b^	1 Firearm unlocked and loaded: sensitivity = 28.2%; specificity = 88.4%
			.03 vs B^b^	
			.17 vs C	
Method D, No. of firearms unlocked or loaded	1.29 (1.06-1.56)	61.6 (56.8-66.5)	.03 vs 1^b^	2 Firearms unlocked or loaded: sensitivity = 51.5%; specificity = 68.4%
			<.01 vs 2^b^	
			.17 vs 3	

Abbreviations: AUROC, area under the receiver operating characteristic curve; OR, odds ratio.

^a^ Results are from unadjusted logistic regression models using perceived teen firearm access as the outcome. *P* values are from the comparison of AUROCs between the different firearm measures (ie, loaded, unlocked, etc).

^b^ Statistical significance at α = .05.

The number of firearms stored unlocked performed the best in estimating teen perceived firearm access, although performance was weak (AUROC, 65.7; 95% CI, 61.4-70.1) (Table 3). The optimum threshold for classifying teens as having access (vs having no access) was storing at least 1 firearm unlocked; information about storing additional firearms unlocked did not improve performance. Although storing 1 or more firearms unlocked performed the best out of all considered measures and thresholds, this measure’s sensitivity and specificity were poor within the full analytic sample (sensitivity: 58%; specificity: 72%). Of teens whose parents reported securely storing all firearms (ie, locked and unloaded), 36.3% (95% CI, 23.6%-49.0%) indicated being able to access a household firearm. Sensitivity analyses with alternative definitions of teen perceived firearm access (ie, access in less than 2 hours or less than 1 hour) found similar patterns for the results (eTables 3 and 4 in Supplement 1).

Storing at least 1 firearm unlocked remained a top-performing classifier of perceived teen firearm access—but with weak estimative ability—among all stratified samples, including male teens, female teens, teens living in metropolitan areas, teens living in nonmetropolitan areas, teens whose parents attained up to a 2-year college degree, and teens whose parents attained at least a 4-year college degree (Figure and eTables 5 to 10 in Supplement 1). Across most demographic strata, the optimum threshold for classifying teens as having perceived access was storing at least 1 firearm unlocked.

**Figure.**
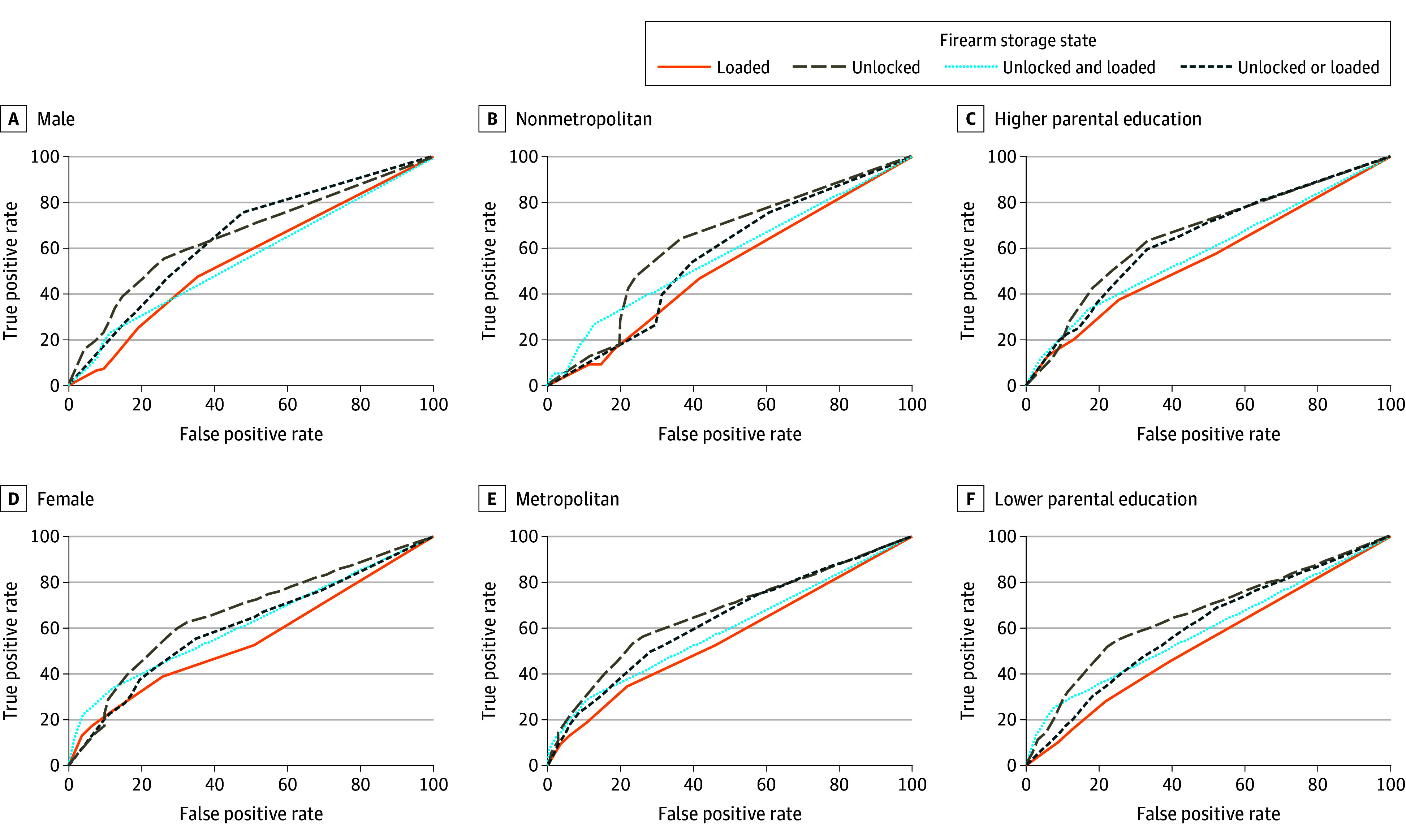
Receiver Operating Characteristic Curves for Firearm Storage Behaviors in Estimating Teen Perceived Firearm Access Across Demographic Strata

Analyses did not find associations between unsecured firearm storage behaviors and perceived teen firearm access after restricting the sample to parent-teen dyads who had at least 1 firearm stored in these respective manners (Table 4). Specifically, analyses did not find that storing more than 1 firearm unlocked (OR, 0.99; 95% CI, 0.74-1.35; n = 215), loaded (OR, 1.30; 95% CI, 0.94-1.83; n = 234), unlocked and loaded (OR, 1.12; 95% CI, 0.67-1.89; n = 100), or unlocked or loaded (OR, 1.18; 95% CI, 0.91-1.53; n = 328) associated with increased the likelihood of perceived teen firearm access (Table 4).

**Table 4.** Associations of Parent-Reported Firearm Storage Behaviors With Teen Perceived Firearm Access Among Households With at Least 1 Firearm Stored in the Respective State^a^

Firearm storage behaviors	OR (95% CI)
No. of firearms unlocked, n = 215	0.99 (0.74-1.35)
No. of firearms loaded, n = 234	1.30 (0.94-1.83)
No. of firearms unlocked and loaded, n = 100	1.12 (0.67-1.89)
No. of firearms unlocked or loaded, n = 328	1.18 (0.91-1.53)

Abbreviation: OR, odds ratio.

^a^ Results are from unadjusted logistic regression models using teen perceived firearm access as the outcome.

## Discussion

More than half of US teens living with firearm-owning parents reported they believed they could obtain and load a firearm on their property. This proportion remained high, at over one-third, when restricting to teens whose parents reported securely storing all firearms (ie, locked and unloaded). These findings are consistent with other recent studies.^17,18^ Specifically, Salhi et al^17^ found that in homes where parents reported storing all firearms locked, nearly one-fourth of adolescents reported they believed they could access a loaded firearm in less than 5 minutes. A separate study found that 40% of US adolescents aged 13 to 18 years reported easy access to and the ability to shoot that firearm, and were more likely to be older, male, non-Hispanic White, and living in high-income households.^18^

These findings provide preliminary support for the harm elimination hypothesis in efforts to improve prevention efforts around unsupervised teen firearm access. Storing at least 1 firearm unlocked was a top-performing (although weak) classifier of teen perceived firearm access among all stratified samples, and information about storing more than 1 firearm unlocked did not improve its estimative ability. While many of the current firearm storage guidelines recommend a dual or triple action to securely storing firearms (ie, locked, unloaded, and ammunition stored separately),^11,19,20^ our findings indicate locked status (regardless of loaded or unloaded status) is the most strongly associated with perceived teen firearm access. Although locking status and ammunition storage may not be as strongly estimative of teens’ perceptions of firearm access, these storage features may influence actual access. Alternatively, it may be that locking status and ammunition storage have little additional influence on teen safety in a house where at least 1 firearm already is stored unlocked.

In further support of the harm elimination hypothesis, associations between the number of unsecured firearms and teen perceptions about access did not appear after restricting the sample to parent-teen dyads who stored 1 or more firearms unsecured. This suggests that the associations between the number of unsecured firearms and teen perceived access found in the full sample may be driven by the differences between families who store no firearms vs 1 firearm unsecured, and not by differences between families who store 1 vs 2 or more firearms unsecured. Together, these findings support the idea that efforts to increase the number of household firearms stored locked and unloaded, while critical to firearm safety efforts, may also be unlikely to prevent teen firearm access for teens living in households with firearms, especially if at least 1 firearm remains unsecured.

### Limitations

This study has limitations. Even as a large, nationally representative study, small sample sizes within specific demographic strata precluded subanalyses among racial and ethnic groups, gender diverse teens, and more granular urbanicity strata. For the variable used for urbanicity (ie, RUCA classification), we acknowledge there could be a lot of potential variability between these largely aggregate groups that could nondifferentially bias estimates. Although the reported response rate was relatively low (31% for parent sample; 21% for teen sample), it included a denominator of noneligible individuals (ie, parents with children outside of age range of 14 to 18 years) as part of the general population, which likely misrepresents the nonresponse rate. For the exposure, firearm storage was parent-reported and subject to social desirability bias leading to a potential underreporting of certain firearm storage behaviors (eg, unlocked and loaded). We also operationalized parent-reported firearm storage behaviors as static. Yet, firearm storage behaviors are typically dynamic, for example, when in use (eg, hunting season), cleaning, or transporting. We were also limited to only firearm storage and did not have additional details on ammunition and its storage location. For the outcome, teen perceived access to firearms is self-reported and is subject to similar biases. Notably, the pathway from perceived firearm access to obtaining actual access and potentially unauthorized firearm use is unclear. However, researchers have demonstrated that self-report has a high reliability and validity for assessing risky behaviors including firearm access.^21,22^ It is also important to note that the outcome, teen perceived firearm access, included an ability to load the firearm once accessed, and therefore likely underestimates any level of access to firearms (ie, can access but cannot load the firearm). Lastly, it is important to note that the sample only included teenaged children (aged 14 to 18 years) and is not representative of US households with younger children.

## Conclusions

Household firearm storage behaviors in the US were a poor estimator for teen perceived firearm access, regardless of teen gender, urbanicity, and parental education. Our results indicated that US teens were more likely to report being able to obtain and load a firearm if they lived in a household with at least 1 unsecured as compared with secured firearms. These findings suggest that when promoting secure storage practices, it should be emphasized that all firearms in the household will need to be stored locked at the very least to reduce teen access. Moreover, the finding that parent-reported firearm storage behavior was a weak estimator of teen firearm access suggests that other factors may be more important estimators of teens’ perceived access. Strictly focusing safety efforts on secure firearm storage may be insufficient to keep teens from accessing firearms unsupervised by adults or parents.

## Notes

Supplement 1. **eFigure. **Flow Diagram for the Analytic Sample and Subsamples **eTable 1. **Select Items From the Parent FACTS National Survey, 2020 **eTable 2. **Select Items From the Teen FACTS National Survey, 2020 **eTable 3**. Comparison of the Accuracy of Parent-Reported Firearm Storage for Estimating Teen-Reported Firearm Access in Less Than 2 Hours Among a Nationally Representative Sample of Adult Firearm Owners and Their Teen Children **eTable 4**. Comparison of the Accuracy of Parent-Reported Firearm Storage for Estimating Teen Percevied Firearm Access in Less Than 1 Hour Among a Nationally Representative Sample of Adult Firearm Owners and Their Teen Children **eTable 5**. Comparison of the Accuracy of Parent-Reported Firearm Storage for Estimating Teen Percieved Firearm Access Among Male Teens **eTable 6**. Comparison of the Accuracy of Parent-Reported Firearm Storage for Estimating Teen Perceived Firearm Access Among Female Teens **eTable 7**. Comparison of the Accuracy of Parent-Reported Firearm Storage for Estimating Teen Perceived Firearm Access Among Youth Living in Metropolitan Areas **eTable 8**. Comparison of the Accuracy of Parent-Reported Firearm Storage for Estimating Teen Perceived Firearm Access Among Youth Living in Nonmetropolitan Areas **eTable 9**. Comparison of the Accuracy of Parent-Reported Firearm Storage for Predicting Teen Perceived Firearm Access Among Youth Whose Parents Attained Up to a 2-Year College Degree **eTable 10**. Comparison of the Accuracy of Parent-Reported Firearm Storage for Estimating Teen Perceived Firearm Access Among Youth Whose Parents Attained at Least a 4-Year College Degree

Supplement 2.
Data Sharing Statement

## References

[1] Lee LK, Douglas K, Hemenway D. Crossing lines—a change in the leading cause of death among US children. N Engl J Med. 2022;386(16):1485-1487. doi:10.1056/NEJMp2200169 35426978

[2] Goldstick JE, Cunningham RM, Carter PM. Current causes of death in children and adolescents in the United States. N Engl J Med. 2022;386(20):1955-1956. doi:10.1056/NEJMc2201761 35443104 PMC10042524

[3] Johnson RM, Barber C, Azrael D, Clark DE, Hemenway D. Who are the owners of firearms used in adolescent suicides? Suicide Life Threat Behav. 2010;40(6):609-611. doi:10.1521/suli.2010.40.6.609 21198329 PMC3085447

[4] Vaishnav A, Smith GA, Badeti J, Michaels NL. An epidemiological study of unintentional pediatric firearm fatalities in the USA, 2009-2018. Inj Epidemiol. 2023;10(1):25. doi:10.1186/s40621-023-00438-5 37357309 PMC10291813

[5] Kellermann AL, Somes G, Rivara FP, Lee RK, Banton JG. Injuries and deaths due to firearms in the home. J Trauma. 1998;45(2):263-267. doi:10.1097/00005373-199808000-00010 9715182

[6] Miller M, Zhang W, Rowhani-Rahbar A, Azrael D. Child access prevention laws and firearm storage: results from a national survey. Am J Prev Med. 2022;62(3):333-340. doi:10.1016/j.amepre.2021.09.016 35190100

[7] Rowhani-Rahbar A, Simonetti JA, Rivara FP. Effectiveness of interventions to promote safe firearm storage. Epidemiol Rev. 2016;38(1):111-124. doi:10.1093/epirev/mxv006 26769724

[8] Monuteaux MC, Azrael D, Miller M. Association of increased safe household firearm storage with firearm suicide and unintentional death among US youths. JAMA Pediatr. 2019;173(7):657-662. doi:10.1001/jamapediatrics.2019.1078 31081861 PMC6515586

[9] Grossman DC, Mueller BA, Riedy C, . Gun storage practices and risk of youth suicide and unintentional firearm injuries. JAMA. 2005;293(6):707-714. doi:10.1001/jama.293.6.707 15701912

[10] Carter PM, Cunningham RM. Clinical approaches to the prevention of firearm-related injury. N Engl J Med. 2024;391(10):926-940. doi:10.1056/NEJMra2306867 39259896

[11] Rowhani-Rahbar A. Firearm storage practices-what constitutes safe? JAMA Netw Open. 2023;6(3):e231452. doi:10.1001/jamanetworkopen.2023.1452 36862417

[12] Berrigan J, Azrael D, Miller M. The number and type of private firearms in the United States. Ann Am Acad Pol Soc Sci. 2022;704(1):70-90. doi:10.1177/00027162231164855

[13] Institute for Firearm Injury Prevention, University of Michigan. Firearm Safety Among Children and Teens (FACTS): multidisciplinary research training program. Accessed November 13, 2024. https://firearminjury.umich.edu/project/firearm-safety-among-children-and-teens-facts-multi-disciplinary-research-training-program/

[14] von Elm E, Altman DG, Egger M, Pocock SJ, Gøtzsche PC, Vandenbroucke JP; STROBE Initiative. The Strengthening the Reporting of Observational Studies in Epidemiology (STROBE) statement: guidelines for reporting observational studies. Lancet. 2007;370(9596):1453-1457. doi:10.1016/S0140-6736(07)61602-X 18064739

[15] Pear VA, McCort CD, Kravitz-Wirtz N, Shev AB, Rowhani-Rahbar A, Wintemute GJ. Risk factors for assaultive reinjury and death following a nonfatal firearm assault injury: a population-based retrospective cohort study. Prev Med. 2020;139:106198. doi:10.1016/j.ypmed.2020.106198 32652134

[16] American Statistical Association statement on statistical significance and *P* values. American Statistical Association. Accessed May 6, 2025. https://www.amstat.org/asa/files/pdfs/P-ValueStatement.pdf

[17] Salhi C, Azrael D, Miller M. Parent and adolescent reports of adolescent access to household firearms in the United States. JAMA Netw Open. 2021;4(3):e210989. doi:10.1001/jamanetworkopen.2021.0989 33687444 PMC7944379

[18] Simonetti JA, Mackelprang JL, Rowhani-Rahbar A, Zatzick D, Rivara FP. Psychiatric comorbidity, suicidality, and in-home firearm access among a nationally representative sample of adolescents. JAMA Psychiatry. 2015;72(2):152-159. doi:10.1001/jamapsychiatry.2014.1760 25548879

[19] Safe storage of firearms. American Academy of Pediatrics. Accessed September 10, 2024. https://www.aap.org/en/advocacy/state-advocacy/safe-storage-of-firearms/?srsltid=AfmBOopLGFfNNfe0WJxR7fNjVufhNfXsJMfpU8--FjZCESmyXY8Y1lDB

[20] Safe storage of firearms: unload it, lock it, store it. United States Department of Justice. Accessed March 10, 2025. https://www.justice.gov/media/1337981/dl?inline

[21] Turner CF, Ku L, Rogers SM, Lindberg LD, Pleck JH, Sonenstein FL. Adolescent sexual behavior, drug use, and violence: increased reporting with computer survey technology. Science. 1998;280(5365):867-873. doi:10.1126/science.280.5365.867 9572724

[22] Tourangeau R, Yan T. Sensitive questions in surveys. Psychol Bull. 2007;133(5):859-883. doi:10.1037/0033-2909.133.5.859 17723033

