# Supplementary material for: Parental Firearm Storage and Their Teens’ Perceived Firearm Access in US Households
Source: JAMA Netw Open. 2025 Jun 10;8(6):e2514443. doi: 10.1001/jamanetworkopen.2025.14443 (PMC12152701; doi:10.1001/jamanetworkopen.2025.14443)
Supplement: Supplement 1. — eFigure. Flow Diagram for the Analytic Sample and Subsamples eTable 1. Select Items From the Parent FACTS National Survey, 2020 eTable 2. Select Items From the Teen FACTS National Survey, 2020 eTable 3. Comparison of the Accuracy of Parent-Reported Firearm Storage for Estimating Teen-Reported Firearm Access in Less Than 2 Hours Among a Nationally Representative Sample of Adult Firearm Owners and Their Teen Children eTable 4. Comparison of the Accuracy of Parent-Reported Firearm Storage for Estimating Teen Percevied Firearm Access in Less Than 1 Hour Among a Nationally Representative Sample of Adult Firearm Owners and Their Teen Children eTable 5. Comparison of the Accuracy of Parent-Reported Firearm Storage for Estimating Teen Percieved Firearm Access Among Male Teens eTable 6. Comparison of the Accuracy of Parent-Reported Firearm Storage for Estimating Teen Perceived Firearm Access Among Female Teens eTable 7. Comparison of the Accuracy of Parent-Reported Firearm Storage for Estimating Teen Perceived Firearm Access Among Youth Living in Metropolitan Areas eTable 8. Comparison of the Accuracy of Parent-Reported Firearm Storage for Estimating Teen Perceived Firearm Access Among Youth Living in Nonmetropolitan Areas eTable 9. Comparison of the Accuracy of Parent-Reported Firearm Storage for Predicting Teen Perceived Firearm Access Among Youth Whose Parents Attained Up to a 2-Year College Degree eTable 10. Comparison of the Accuracy of Parent-Reported Firearm Storage for Estimating Teen Perceived Firearm Access Among Youth Whose Parents Attained at Least a 4-Year College Degree [file jamanetwopen-e2514443-s001.pdf]

Supplemental Online Content

Hastings KG, Carter PM, Zimmerman M, Sokol R. Parental firearm storage and their teens' perceived firearm access in the US. *JAMA Netw Open*. 2025;8(6):e2514443. doi:10.1001/jamanetworkopen.2025.14443

eFigure 1. Flow Diagram for the Analytic Sample and Subsamples

eTable 1. Select Items From the Parent FACTS National Survey, 2020

eTable 2. Select Items From the Teen FACTS National Survey, 2020

eTable 3. Comparison of the Accuracy of Parent-Reported Firearm Storage for Estimating Teen-Reported Firearm Access in Less Than 2 Hours Among a Nationally Representative Sample of Adult Firearm Owners and Their Teen Children

eTable 4. Comparison of the Accuracy of Parent-Reported Firearm Storage for Estimating Teen Percevied Firearm Access in Less Than 1 Hour Among a Nationally Representative Sample of Adult Firearm Owners and Their Teen Children

eTable 5. Comparison of the Accuracy of Parent-Reported Firearm Storage for Estimating Teen Percieved Firearm Access Among Male Teens

eTable 6. Comparison of the Accuracy of Parent-Reported Firearm Storage for Estimating Teen Perceived Firearm Access Among Female Teens

eTable 7. Comparison of the Accuracy of Parent-Reported Firearm Storage for Estimating Teen Perceived Firearm Access Among Youth Living in Metropolitan Areas

eTable 8. Comparison of the Accuracy of Parent-Reported Firearm Storage for Estimating Teen Perceived Firearm Access Among Youth Living in Nonmetropolitan Areas

eTable 9. Comparison of the Accuracy of Parent-Reported Firearm Storage for Predicting Teen Perceived Firearm Access Among Youth Whose Parents Attained Up to a 2-Year College Degree

eTable 10. Comparison of the Accuracy of Parent-Reported Firearm Storage for Estimating Teen Perceived Firearm Access Among Youth Whose Parents Attained at Least a 4-Year College Degree

This supplemental material has been provided by the authors to give readers additional information about their work.

© 2025 Hastings KG et al. *JAMA Network Open*.

© 2025 Hastings KG et al. *JAMA Network Open*.

eFigure 1. Flow diagram for the analytic sample and sub-samples.

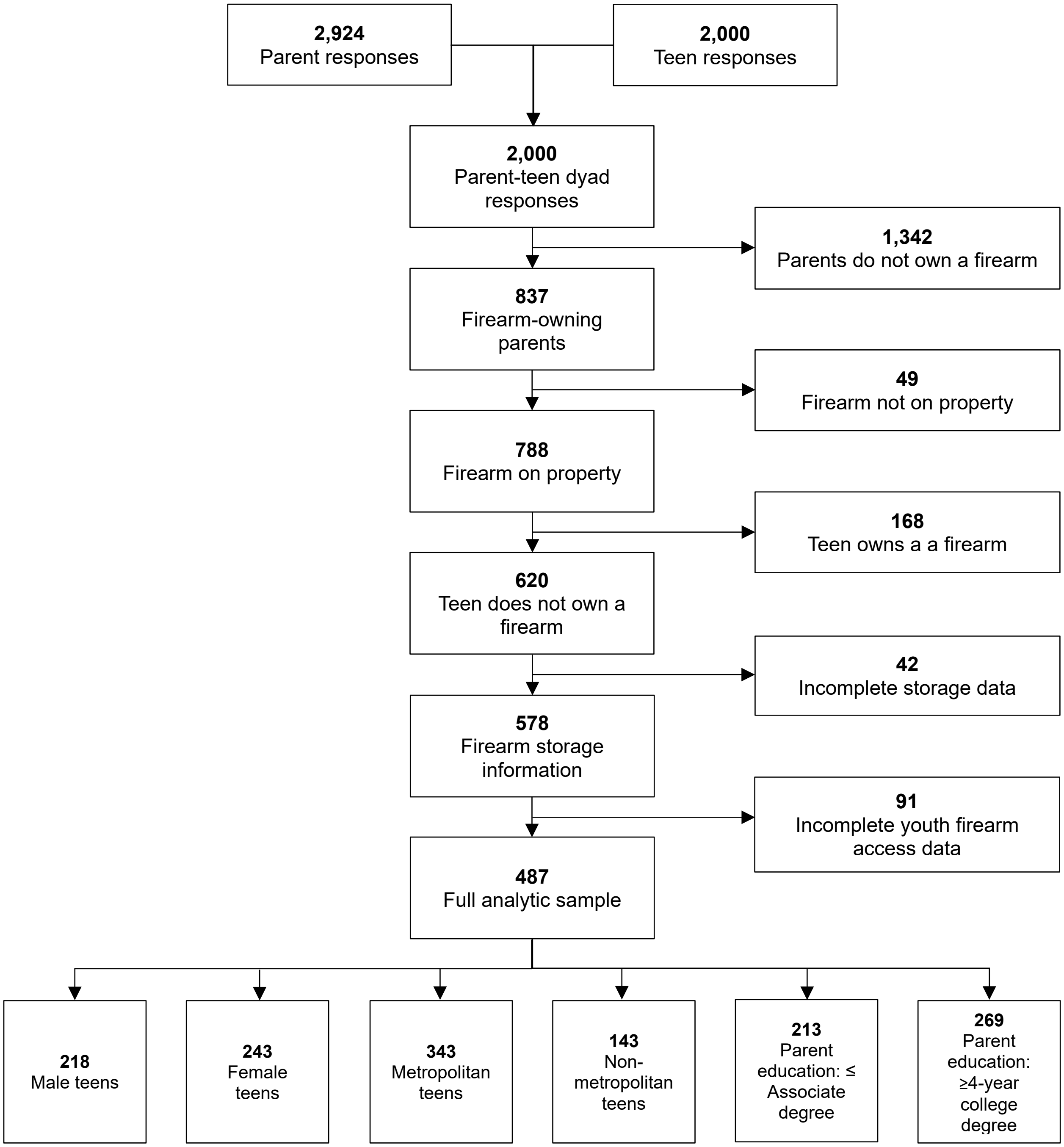

© 2025 Hastings KG et al. *JAMA Network Open*.

eTable 1. Select Items From the Parent FACTS National Survey, 2020

Domain	Question	Response	Coding
Firearm ownership	Do you personally own a gun? Please exclude guns that belong to people you live with.	1=Yes<br>2=No	**Firearm owner** = 1<br>**Firearm non-owner** = 2
Firearm storage state	Of the handguns on your property that you personally own, how many are kept in the following ways?<br>Loaded & locked<br>Loaded & unlocked<br>Unloaded & locked<br>Unloaded & unlocked	[Number box]	*Each capped at 5*<br><br>**Number loaded** =<br>(# handguns loaded & locked) +<br>(# long guns loaded & locked) +<br>(# handguns loaded & unlocked) +<br>(# long guns loaded & unlocked)<br><br>**Number unlocked =**<br>(# handguns loaded & unlocked) +<br>(# long guns loaded & unlocked) +<br>(# handguns unloaded & unlocked) +<br>(# long guns unloaded & unlocked)<br><br>**Number loaded OR unlocked =**<br>(# handguns loaded & locked) +<br>(# long guns loaded & locked) +<br>(# handguns loaded & unlocked) +<br>(# long guns loaded & unlocked) +<br>(# handguns unloaded & unlocked) +<br>(# long guns unloaded & unlocked)<br><br>**Number loaded & unlocked =**<br>(# handguns loaded & unlocked) +<br>(# long guns loaded & unlocked)
	Of the long guns on your property that you personally own, how many are kept in the following ways?<br>Loaded & locked<br>Loaded & unlocked<br>Unloaded & locked<br>Unloaded & unlocked	[Number box]	
Age	[Panel member age, provided by Gallup]	Age, in years	Age, in years
Gender	What is your gender?	1=Male<br>2=Female	**Male** = 1<br>**Female** = 2
Race and ethnicity	Which of the following describes your race? You may select one or more.	1=White<br>2=Other<br>3=Black<br>4=Asian<br>5=Hispanic	**White** = 1<br>**Another race not listed** = 2<br>**Black** = 3<br>**Asian** = 4<br>**Hispanic** = 5
Education level	What is the highest level of school you have completed or the highest degree you have received?	1= Less than high school<br>2= High school graduate<br>3= Technical, trade, vocational, or business school or program<br>4= Some college, no degree<br>5= Two-year associate degree<br>6= Four-year bachelor's degree<br>7= Some postgraduate or professional schooling, no degree<br>8= Postgraduate or professional degree	**Less than high school through associate degree** = 1 | 2 | 3 | 4 | 5<br><br>**4-year degree or more** = 6 | 7 | 8

© 2025 Hastings KG et al. *JAMA Network Open*.

eTable 2. Select Items From the Teen FACTS National Survey, 2020

Domain	Question	Response	Coding
Firearm access	How long would it take you to get one of the guns on your property and load it if it wasn't already loaded?	1=Less than 5 minutes<br>2=Less than an hour<br>3=Less than two hours<br>4=More than two hours<br>5=I couldn't get access to a gun on my property and load it	**Firearm access =** 1 | 2 | 3 | 4<br>**No firearm access =** 5
Age	How old are you?	14=14<br>15=15<br>16=16<br>17=17<br>18=18	**14**=14<br>**15**=15<br>**16**=16<br>**17**=17<br>**18**=18
Gender	What is your current gender identity?	1=Female<br>2=Male<br>3=Trans Male/Trans Man<br>4=Trans Female/Trans Woman<br>5=Genderqueer/Gender non-conforming<br>6=Other identity, please specify	**Male** = 1<br>**Female** = 2<br>**Gender expansive** = 3 | 4 | 5 |6
Race	Which of the following best describes your racial background?	1=Black or African American<br>2=White<br>3=Native Hawaiian/Other Pacific Islander<br>4=American Indian/Alaska Native<br>5=Asian<br>6=Middle Eastern/North African<br>7=Unknown/Other<br>8=Multi-Racial	**Black or African American** = 1<br>**White** = 2<br>**Native Hawaiian/Other Pacific Islander** = 3<br>**American Indian/Alaska Native** = 4<br>**Asian** = 5<br>**Middle Eastern/North African** = 6<br>**Unknown/Other** = 7<br>**Multi-Racial** = 8
Ethnicity	Are you of Hispanic, Latino, or Spanish origin -- such as Mexican, Puerto Rican, Cuban, or other Spanish origin?	1 = Yes<br>2 = No	**Less than high school through associate degree** = 1 | 2 | 3 | 4 | 5<br><br>**4-year degree or more** = 6 | 7 | 8

© 2025 Hastings KG et al. *JAMA Network Open*.

eTable 3. Comparison of the Accuracy of Parent-Reported Firearm Storage for Estimating Teen-Reported Firearm Access in Less Than 2 Hours Among a Nationally Representative Sample of Adult Firearm Owners and Their Teen Children (n = 487)

Firearm storage state	OR (95% CI)	AUC (95% CI)	Optimum threshold	*p*-value
1. # of firearms unlocked	1.29 (1.04, 1.60)	66.0 (61.7, 70.4)	1 firearm unlocked: sensitivity = 58.8%, specificity = 70.7%	**<0.01 vs 2**<br>**<0.01 vs 3**<br>0.07 vs 4
2. # of firearms loaded	1.29 (1.05, 1.59)	55.9 (51.2, 60.6)	2 firearms loaded: sensitivity = 33.5%, specificity =77.3%	**<0.01 vs 1**<br>0.09 vs 3<br>**<0.01 vs 4**
3. # of firearms unlocked & loaded	1.46 (1.01, 2.11)	59.2 (55.7, 62.7)	1 firearm unlocked & loaded: sensitivity = 29.4%, specificity = 88.4%	**<0.01 vs 1**<br>0.09 vs 2<br>0.11 vs 4
4. # of firearms unlocked OR loaded	1.30 (1.08, 1.58)	62.7 (57.9, 67.5)	2 firearms unlocked *or* loaded: sensitivity = 53.1%, specificity = 68.6%	0.07 vs 1<br>**>0.01 vs 2**<br>0.11 vs 3

Notes. Results are from unadjusted logistic regression models using perceived teen firearm access as the outcome. *p*-values are from the comparison of AUCs between the different firearm measures (i.e., loaded, unlocked, etc.). **Bold** indicates significance at α = 0.05. Abbreviations: AUC, area under the receiver operating characteristic curve; CI, confidence interval; OR, odds ratio.

© 2025 Hastings KG et al. *JAMA Network Open*.

eTable 4. Comparison of the Accuracy of Parent-Reported Firearm Storage for Estimating Teen Percevied Firearm Access in Less Than 1 Hour Among a Nationally Representative Sample of Adult Firearm Owners and Their Teen Children (n = 487)

Firearm storage state	OR (95% CI)	AUC (95% CI)	Optimum threshold	*p*-value
1. # of firearms unlocked	1.28 (1.04, 1.58)	64.9 (60.4, 69.3)	1 firearm unlocked: sensitivity = 58.4%, specificity = 68.9%	**<0.01 vs 2**<br>**<0.01 vs 3**<br>0.11 vs 4
2. # of firearms loaded	1.31 (1.06, 1.61)	56.5 (51.8, 61.2)	2 firearms loaded: sensitivity = 34.3%, specificity =77.6%	**<0.01 vs 1**<br>0.08 vs 3<br>**<0.01 vs 4**
3. # of firearms unlocked & loaded	1.45 (1.02, 2.05)	59.8 (56.3, 63.4)	1 firearm unlocked *&* loaded: sensitivity = 30.4%, specificity = 88.6%	**<0.01 vs 1**<br>0.08 vs 2<br>0.34 vs 4
4. # of firearms unlocked OR loaded	1.32 (1.09, 1.59)	62.0 (57.1, 66.8)	2 firearms unlocked *or* loaded: sensitivity = 52.8%, specificity = 67.3%	0.11 vs 1<br>**<0.01 vs 2**<br>0.34 vs 3

Notes. Results are from unadjusted logistic regression models using teen perceived firearm access as the outcome. *p*-values are from the comparison of AUCs between the different firearm measures (i.e., loaded, unlocked, etc.). **Bold** indicates significance at α = 0.05. Abbreviations: AUC, area under the receiver operating characteristic curve; CI, confidence interval; OR, odds ratio.

© 2025 Hastings KG et al. *JAMA Network Open*.

eTable 5. Comparison of the Accuracy of Parent-Reported Firearm Storage for Estimating Teen Percieved Firearm Access Among Male Teens (n = 218)

Firearm storage behaviors	OR (95% CI)	AUC (95% CI)	Optimum threshold	*p*-value
1. # of firearms unlocked	1.32 (0.96, 1.83)	65.7 (59.4, 72.1)	1 firearm unlocked: sensitivity = 55.3%, specificity = 74.0%	**0.01 vs 2**<br>**<0.01 vs 3**<br>0.71 vs 4
2. # of firearms loaded	1.20 (0.86, 1.69)	55.3 (48.5, 62.2)	1 firearm loaded: sensitivity = 47.4%, specificity = 64.4%	**0.01 vs 1**<br>0.96 vs 3<br>**<0.01 vs 4**
3. # of firearms unlocked & loaded	1.11 (0.72, 1.72)	55.5 (50.6, 60.4)	1 firearm unlocked & loaded: sensitivity = 21.9%, specificity = 89.4%	**<0.01 vs 1**<br>0.96 vs 2<br>**<0.01 vs 4**
4. # of firearms unlocked OR loaded	1.45 (1.07, 1.96)	64.8 (57.7, 71.8)	1 firearm unlocked *or* loaded: sensitivity = 75.4%, specificity = 51.9%	0.71 vs 1<br>**<0.01 vs 2**<br>**<0.01 vs 3**

Notes. Results are from unadjusted logistic regression models using teen perceived firearm access as the outcome. *p*-values are from the comparison of AUCs. **Bold** indicates significance at α = 0.05. Abbreviations: AUC, area under the receiver operating characteristic curve; CI, confidence interval; OR, odds ratio.

© 2025 Hastings KG et al. *JAMA Network Open*.

eTable 6. Comparison of the Accuracy of Parent-Reported Firearm Storage for Estimating Teen Perceived Firearm Access Among Female Teens (n = 243)

Firearm storage behaviors	OR (95% CI)	AUC (95% CI)	Optimum threshold	*p*-value
1. # of firearms unlocked	1.25 (0.93, 1.69)	66.2 (59.9, 72.5)	1 firearm unlocked: sensitivity = 61.5%, specificity = 69.4%	**<0.01 vs 2**<br>0.11 vs 3<br>**0.03 vs 4**
2. # of firearms loaded	1.34 (1.04, 1.73)	54.9 (48.2, 61.7)	2 firearms loaded: sensitivity = 38.5%, specificity = 74.1%	**<0.01 vs 1**<br>**0.01 vs 3**<br>**0.04 vs 4**
3. # of firearms unlocked & loaded	2.44 (1.34, 4.43)	61.7 (56.6, 66.8)	1 firearm unlocked & loaded: sensitivity = 34.8%, specificity = 87.0%	0.11 vs 1<br>**0.01 vs 2**<br>0.72 vs 4
4. # of firearms unlocked OR loaded	1.18 (0.92, 1.52)	60.5 (53.6, 67.5)	2 firearms unlocked *or* loaded: sensitivity = 55.6%, specificity = 64.8%	**0.03 vs 1**<br>**0.04 vs 2**<br>0.72 vs 3

Notes. Results are from unadjusted logistic regression models using teen perceived firearm access as the outcome. *p*-values are from the comparison of AUCs. **Bold** indicates significance at α = 0.05. Abbreviations: AUC, area under the receiver operating characteristic curve; CI, confidence interval; OR, odds ratio.

© 2025 Hastings KG et al. *JAMA Network Open*.

eTable 7. Comparison of the Accuracy of Parent-Reported Firearm Storage for Estimating Teen Perceived Firearm Access Among Youth Living in Metropolitan Areas (n = 343)

Firearm storage behaviors	OR (95% CI)	AUC (95% CI)	Optimum threshold	*p*-value
1. # of firearms unlocked	1.38 (1.02, 1.87)	66.5 (61.5, 71.6)	1 firearm unlocked: sensitivity = 55.6%, specificity = 75.2%	**<0.01 vs 2**<br>**<0.01 vs 3**<br>0.15 vs 4
2. # of firearms loaded	1.36 (1.04, 1.79)	55.7 (50.1, 61.3)	2 firearms loaded: sensitivity = 34.8%, specificity = 78.2%	**<0.01 vs 1**<br>0.14 vs 3<br>**<0.01 vs 4**
3. # of firearms unlocked & loaded	1.49 (0.90, 2.46)	59.2 (55.1, 63.3)	1 firearm unlocked *&* loaded: sensitivity = 28.7%, specificity = 89.1%	**<0.01 vs 1**<br>0.14 vs 2<br>0.13 vs 4
4. # of firearms unlocked OR loaded	1.42 (1.10, 1.84)	63.2 (57.5, 68.9)	2 firearms unlocked *or* loaded: sensitivity = 50.0%, specificity = 71.5%	0.15 vs 1<br>**<0.01 vs 2**<br>0.13 vs 3

Notes. Results are from unadjusted logistic regression models using teen perceived firearm access as the outcome. *p*-values are from the comparison of AUCs. **Bold** indicates significance at α = 0.05. Abbreviations: AUC, area under the receiver operating characteristic curve; CI, confidence interval; OR, odds ratio.

© 2025 Hastings KG et al. *JAMA Network Open*.

eTable 8. Comparison of the Accuracy of Parent-Reported Firearm Storage for Estimating Teen Perceived Firearm Access Among Youth Living in Nonmetropolitan Areas (n = 143)

Firearm storage behaviors	OR (95% CI)	AUC (95% CI)	Optimum threshold	*p*-value
1. # of firearms unlocked	1.09 (0.79, 1.50)	62.5 (53.6, 71.5)	1 firearm unlocked: sensitivity = 63.9%, specificity = 63.3%	**0.04 vs 2**<br>0.16 vs 3<br>0.06 vs 4
2. # of firearms loaded	1.13 (0.82, 1.58)	51.5 (42.6, 60.3)	1 firearms loaded: sensitivity = 47.0%, specificity = 58.3%	**0.04 vs 1**<br>0.11 vs 3<br>0.18 vs 4
3. # of firearms unlocked & loaded	1.37 (0.86, 2.18)	57.0 (50.4, 63.6)	1 firearm unlocked & loaded: sensitivity = 27.7%, specificity = 86.7%	0.16 vs 1<br>0.11 vs 2<br>0.88 vs 4
4. # of firearms unlocked OR loaded	1.07 (0.79, 1.45)	56.3 (46.7, 65.9)	1 firearm unlocked *or* loaded: sensitivity = 74.7%, specificity = 40.0%	0.06 vs 1<br>0.18 vs 2<br>0.88 vs 3

Notes. Results are from unadjusted logistic regression models using teen perceived firearm access as the outcome. *p*-values are from the comparison of AUCs. **Bold** indicates significance at α = 0.05. Abbreviations: AUC, area under the receiver operating characteristic curve; CI, confidence interval; OR, odds ratio.

© 2025 Hastings KG et al. *JAMA Network Open*.

eTable 9. Comparison of the Accuracy of Parent-Reported Firearm Storage for Predicting Teen Perceived Firearm Access Among Youth Whose Parents Attained Up to a 2-Year College Degree (n = 213)

Firearm storage state	OR (95% CI)	AUC (95% CI)	Optimum threshold	*p*-value
1. # of firearms unlocked	1.20 (0.93, 1.56)	66.0 (59.1, 72.8)	1 firearm unlocked: sensitivity = 63.5%, specificity = 66.3%	**0.02 vs 2**<br>**<0.01 vs 3**<br>0.55 vs 4
2. # of firearms loaded	1.34 (1.01, 1.77)	55.4 (48.1, 62.7)	2 firearms loaded: sensitivity = 37.4%, specificity = 74.5%	**0.02 vs 1**<br>0.36 vs 3<br>**<0.01 vs 4**
3. # of firearms unlocked & loaded	1.31 (0.90, 1.91)	58.1 (52.3, 63.9)	1 firearm unlocked *&* loaded: sensitivity = 33.0%, specificity = 82.7%	**<0.01 vs 1**<br>0.36 vs 2<br>0.06 vs 4
4. # of firearms unlocked OR loaded	1.33 (1.02, 1.73)	64.3 (57.1, 71.6)	2 firearms unlocked *or* loaded: sensitivity = 60.0%, specificity = 66.3%	0.55 vs 1<br>**<0.01 vs 2**<br>0.06 vs 3

Notes. Results are from unadjusted logistic regression models using teen perceived firearm access as the outcome. *p*-values are from the comparison of AUCs. **Bold** indicates significance at α = 0.05. Abbreviations: AUC, area under the receiver operating characteristic curve; CI, confidence interval; OR, odds ratio.

© 2025 Hastings KG et al. *JAMA Network Open*.

eTable 10. Comparison of the Accuracy of Parent-Reported Firearm Storage for Estimating Teen Perceived Firearm Access Among Youth Whose Parents Attained at Least a 4-Year College Degree (n = 269)

Firearm storage behaviors	OR (95% CI)	AUC (95% CI)	Optimum threshold	*p*-value
1. # of firearms unlocked	1.62 (1.23, 2.14)	65.9 (60.2, 71.5)	1 firearm unlocked: sensitivity = 53.8%, specificity = 76.6%	**<0.01 vs 2**<br>**<0.01 vs 3**<br>**0.02 vs 4**
2. # of firearms loaded	1.15 (0.85, 1.56)	53.5 (47.3, 59.7)	2 firearms loaded: sensitivity = 28.3%, specificity = 78.2%	**<0.01 vs 1**<br>**0.04 vs 3**<br>**<0.01 vs 4**
3. # of firearms unlocked & loaded	2.87 (1.45, 5.69)	59.0 (54.7, 63.2)	1 firearm unlocked *&* loaded: sensitivity = 24.8%, specificity = 92.7%	**<0.01 vs 1**<br>**0.04 vs 2**<br>0.72 vs 4
4. # of firearms unlocked OR loaded	1.22 (0.96, 1.55)	60.0 (53.5, 66.6)	1 firearm unlocked *or* loaded: sensitivity = 69.7%, specificity = 46.8%	**0.02 vs 1**<br>**<0.01 vs 2**<br>0.72 vs 3

Notes. Results are from unadjusted logistic regression models using teen perceived firearm access as the outcome. *p*-values are from the comparison of AUCs. **Bold** indicates significance at α = 0.05. Abbreviations: AUC, area under the receiver operating characteristic curve; CI, confidence interval; OR, odds ratio.

© 2025 Hastings KG et al. *JAMA Network Open*.
