# Supplementary material for: Parental Firearm Storage and Their Teens’ Perceived Firearm Access in US Households
Source: JAMA Netw Open. 2025 Jun 10;8(6):e2514443. doi: 10.1001/jamanetworkopen.2025.14443 (PMC12152701; doi:10.1001/jamanetworkopen.2025.14443)
Supplement: Supplement 2. — Data Sharing Statement [file jamanetwopen-e2514443-s002.pdf]

Data Sharing Statement

Data
Data available: No

Additional Information
Explanation for why data not available: Data is kept confidential according to our Institutional Review Board regulations at the University of Michigan
